# Clinical outcome and prognostic factors for Asian patients in Phase I clinical trials

**DOI:** 10.1038/s41416-023-02193-2

**Published:** 2023-02-16

**Authors:** Jerold Loh, Jiaxuan Wu, Jenny Chieng, Aurora Chan, Wei-Peng Yong, Raghav Sundar, Soo-Chin Lee, Andrea Wong, Joline S. J. Lim, David S. P. Tan, Ross Soo, Boon-Cher Goh, Bee-Choo Tai, Cheng E. Chee

**Affiliations:** 1 Department of Haematology-Oncology, National University Cancer Institute, Singapore, Singapore; 2 Yong Loo Lin School of Medicine, National University of Singapore, Singapore, Singapore; 3 NUS Saw Swee Hock School of Public Health, Singapore, Singapore

**Keywords:** Drug development, Institutions, Cancer immunotherapy, Targeted therapies

## Abstract

**Background:**

Patient selection is key in Phase I studies, and prognosis can be difficult to estimate in heavily pre-treated patients. Previous prognostic models like the Royal Marsden Hospital (RMH) score or using the neutrophil–lymphocyte ratio (NLR) have not been validated in current novel therapies nor in the Asian Phase I population.

**Methods:**

We conducted a retrospective review of 414 patients with solid tumours participating in Phase I studies at our centre between October 2013 and December 2020.

**Results:**

The RMH model showed poorer prognosis with increasing scores [RMH score 1, HR 1.28 (95% CI: 0.96–1.70); RMH score 2, HR 2.27 (95% CI: 1.62–3.17); RMH score 3, HR 4.14 (95% CI: 2.62–6.53)]. NLR did not improve the AUC of the model. Poorer ECOG status (ECOG 1 vs. 0: HR = 1.59 (95% CI = 1.24–2.04), *P* < 0.001) and primary tumour site (GI vs. breast cancer: HR = 3.06, 95% CI = 2.16–4.35, *P* < 0.001) were prognostic.

**Conclusions:**

We developed a NCIS prognostic score with excellent prognostic ability for both short-term and longer-term survival (iAUC: 0.71 [95% CI 0.65–0.76]), and validated the RMH model in the largest Asian study to date.

## Introduction

Our understanding of tumour biology has grown exponentially and a large number of novel molecularly targeted agents and immunotherapy have entered the clinic. Many of these agents have different side effect profiles compared to cytotoxic chemotherapy and their development plan may differ, with an emphasis on understanding target impact. Phase I studies include first-in-human studies as well as studies that combine two or more experimental drugs for the first time and may include patients who have exhausted standard therapies. Their objectives are to evaluate safety, pharmacokinetic and pharmacodynamic properties of these agents; to establish an optimal dose for Phase 2 efficacy trials; to describe initial antitumour response; and to gain information about the effect of a targeted agent on its target [1, 2]. These trials, therefore, form a critical link between the preclinical setting and subsequent definitive trials determining efficacy. How patients fare on such trials and whether there are clinical and tumour characteristics that may influence patient selection, consequently, are important issues of concern [3]. Previous studies have suggested that physicians tend to make overly optimistic estimates of the survival of patients with advanced cancer, so objective parameters may improve prognostic accuracy [4, 5].

A typical inclusion criterion for Phase 1 studies is ‘life expectancy >3 months’, however, the overall survival (OS) of patients with advanced solid malignancies is often difficult to predict, more so in the era of targeted therapy and immunotherapy. Prognostic scores have been developed to identify the prognosis of patients in Phase I studies. The Royal Marsden Hospital (RMH) prognostic score (which incorporates serum albumin, lactate dehydrogenase (LDH) levels, and the number of metastatic sites) is commonly used [6]. The RMH score has been validated to predict OS in Phase 1 trial patients in the Western population [7–10]. With tumour-promoting inflammation recognised as a hallmark of cancer, a high neutrophil–lymphocyte ratio (NLR) has become recognised as a negative prognostic factor as well [11]. However, the cut-off for high NLR has been difficult to define varying between studies and with different types of cancers [11]. The Marsden group also sought to better define and integrate the NLR into a prognostic score for the Phase I population, resulting in the RMH + NLR50 score (using the median NLR for their study population) which had the best discriminative ability [12]. This too was performed in a Western population. Similar risk scores have not been validated in Asian patients who may have biological differences in certain cancer types [13–15]. It is also important to validate the RMH score and NLR for newer Phase I studies, including immunotherapy and vaccine studies, given the rapid evolution of cancer therapy. Our study aims to validate the RMH score and RMH + NLR50 score in an academic Phase 1 clinical trials unit in Singapore and is the largest Asian study to date which includes novel Phase 1 therapies. We also aim to identify other relevant prognostic factors within our study population and develop a prognostic model with improved discriminative ability.

## Methods

### Study design and patient characteristics

We conducted a retrospective review of patients with solid tumours participating in Phase I studies at the National University Cancer Institute, Singapore (NCIS) between October 2013 and December 2020. Patients enrolled in the studies fulfilled the eligibility criteria of the respective studies. Patient demographics, cancer and treatment history, clinical parameters including components of the RMH score and NLR ratio, tumour molecular information and date of death or last follow-up were retrieved from the electronic medical records between October 2013 and December 2020. The RMH score evaluates serum albumin (<35 g/dL constitutes 1 point), number of metastatic sites (3 or more sites constitute 1 point) and serum LDH (more than one-time upper limit normal constitutes 1 point); with scores 0–1 and 2–3 connoting good and poor prognosis, respectively. NLR ratio is calculated by dividing first encounter serum neutrophil count by serum lymphocyte count. The study was approved by the institutional review board.

### Statistical considerations

Categorical variables were summarised based on counts and percentages while continuous variables were described in terms of median and interquartile range (IQR).

Overall survival (OS) duration was measured from the start of therapy to the date of death. Patients who remained alive at the end of study were censored at the date of last follow-up. Survival curves were estimated via the Kaplan–Meier method. To compare survival distributions between groups, the log-rank test was employed. Multivariable Cox regression models were applied to validate the RMH and RMH + NLR50 models for mortality. The effect estimates were quantified based on the hazard ratio (HR) and its 95% confidence interval (CI). The proportional hazards assumption was evaluated using scaled Schoenfeld residuals.

### Model validation

Both the RMH and RMH + NLR50 models were externally validated using the NCIS data, and their performances were evaluated using Harrell’s C-statistic and time-dependent area under the curve (AUC(*t*)) at 3- and 6-month and integrated AUC (iAUC) with *t* ranging from 0.25 to 1.5 years. The AUC(*t*) was estimated using the nearest neighbour method as discussed by Heagerty et al. [16]. To account for the variability of AUC(*t*) and iAUC, 1000 bootstrap replicates were drawn to estimate the standard error and its 95% CI.

### Model update

In addition to the RMH score, the following covariates were considered for the model update: gender, age, ECOG performance status (PS), number of co-morbidities, number of prior therapies, aspartate aminotransferase (AST), platelets, haemoglobin, NLR, tumour classification, and whether they were treated with chemotherapy, immunotherapy, targeted therapy and/or vaccines. Significant variables (*P* < 0.05) at the univariable analysis were considered for further inclusion in the multivariable Cox model, assuming complete case analysis. The final model was obtained via the backward selection procedure. The performance characteristics of the updated NCIS model were further compared with those of the RMH and RMH + NLR50 models. Based on this updated model, the NCIS prognostic score was developed using the method proposed by Sullivan et al. [17], and patients were subsequently classified into low- and high-risk groups based on these scores.

All statistical evaluations were made assuming a two-sided test at the 5% level of significance. Statistical analyses were conducted using STATA version 15.0. In addition, analyses of AUC(*t*) and iAUC were implemented using the survivalROC() and IntAUC() functions of R (version 3.6).

## Results

### Study population

There were 414 patients (157 [38%] male and 257 [62%] female) recruited in 40 Phase I studies at NCIS (Table 1). Most of these patients have good premorbid status with ECOG PS of 0–1 (394 [95%]), nil or one co-morbidity (335 [81%]) and were heavily pre-treated and progressed on at least 3 lines of prior therapy (195 [47%]). The most common cancers in this population were breast cancer (117 [28%]) and gastrointestinal (GI) cancers (colorectal and upper GI cancers) (107 [26%]). Most were enrolled in targeted therapy trials (297 [72%]). With a median follow-up duration of 2.3 years, 283 deaths were observed. The 90-day mortality rate was 17.4%. The median overall survival was 10.1 months. As most studies enrolled only ECOG 0–1 patients, patients with ECOG 2 were excluded from subsequent analysis.

**Table 1:** Baseline and treatment characteristics.

Characteristics	NCIS study population (*n* = 414)		Arkenau et al. [6] (*n* = 212)		Kumar et al. [12] (*n* = 300)	
	No. of pts	%	No. of pts	%	No. of pts	%
Gender						
Male	157	38	142	67	159	53
Female	257	62	70	33	141	47
Median age, years (IQR)	60 [51–66]				58 [49–65]	
ECOG PS						
0	190	46	58	28	104	35
1	204	49	137	66	194	64
2	8	2	13	6	1	1
Number of prior therapies						
0–2	219	53	110	52		
≥3	195	47	102	48		
Number of metastatic sites						
0–2	228	55	135	64	104	35
≥ 3	186	45	77	36	196	65
Metastatic sites						
Liver	194	47	57	27		
Lung	192	46	86	41		
Bone	116	28	62	29		
Albumin, unit						
≥35	332	80	91	57		
<35	82	20	121	43		
LDH, IU/L						
≤580	277	67	108	51		
>580	136	33	104	49		
RMH score						
0	145	35	119	56	70	23
1	162	39			129	43
2	76	18	93	44	93	31
3	30	7			8	3
Median NLR (IQR)	3.39 (2.09–5.04)				3.08 (2.06–4.49)	
Tumour classification Breast	117	28	33^7^	16		
GI (colorectal + upper GI)	107	26	26	12		
Other	190	46	153	72		
Treatment						
Chemotherapy	36	9	64	30		
Targeted therapy	297	72	148	70		
Immunotherapy	74	18				
Vaccines	18	4				

Note: 1. The figures are presented in terms of frequency and percentage, unless otherwise stated.

2. Arkenau et al. [6], 69% of the participants were <65 years, and 31% were ≥65 years.

3. In total, 12 patients in the NCIS study population did not have ECOG PS recorded.

4. Median albumin was 36 (IQR 33–39) in Kumar et al. [12].

5. LDL was presented based on the institutional upper limit of normal i.e., 580 IU/L for NCIS study population and 192 U/L for Arkenau et al. [6]. It was missing for one patient in the NCIS study population.

6. For Arkenau et al. [6], the score for RMH was combined for 0–1 (119, 56%) and 2–3 (93, 44%).

7. For Arkenau et al. [6], breast cancer patients were included in the same group as gynaecological patients.

8. Other cancers for the NCIS population included lymphoma (3.4%), hepatocellular carcinoma (6.3%), head and neck cancers (8.0%), lung cancer (8.2%) and gynaecological cancer (12.8%).

### External validation of the RMH and RMH + NLR50 models using NCIS data

**Fig. 1:**
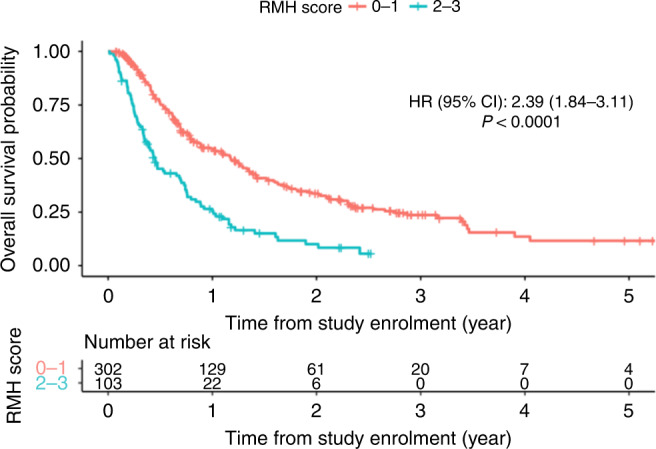
Overall survival according to RMH Score for patients enrolled in Phase I trials.

When applied on the NCIS data, the RMH score was found to be significantly associated with overall survival (Fig. 1 and Table 2). The RMH model showed a clear trend of poorer prognosis with increasing scores [RMH score 1, HR 1.28 (95% CI: 0.96–1.70); RMH score 2, HR 2.27 (95% CI: 1.62–3.17); RMH score 3, HR 4.14 (95% CI: 2.62–6.53)]. The 3-month AUC of the RMH model was 0.72 (95% CI 0.63–0.79) whereas the iAUC was 0.67 (95% CI: 0.61–0.72).

**Table 2:** Validation and performance of Model 1 (RMH) and Model 2 (RMH + NLR50) in comparison with the updated Model 3 (NCIS).

Characteristics	Model 1: RMH		Model 2: RMH + NLR50		Model 3: NCIS	
	HR (95% CI)	*P* value	HR (95% CI)	*P* value	HR (95% CI)	*P* value
RMH score						
0	1		1		1	
1	1.28 (0.96–1.70)	0.099	1.29 (0.96–1.72)	0.087	1.22 (0.91–1.63)	0.189
2	2.27 (1.62–3.17)	<0.001	2.16 (1.54–3.02)	<0.001	2.13 (1.51–3.00)	<0.001
3	4.14 (2.62–6.53)	<0.001	4.05 (2.56–6.39)	<0.001	3.80 (2.40–6.02)	<0.001
NLR50^a^						
≤3.4	–	–	1		–	–
>3.4	–	–	1.38 (1.08–1.76)	0.009	–	–
ECOG PS						
0	–	–	–	–	1	
1	–	–	–	–	1.59 (1.24–2.04)	<0.001
Tumour type						
Breast	–	–	–	–	1	
GI malignancies	–	–	–	–	3.06 (2.16–4.35)	<0.001
Others	–	–	–	–	1.81 (1.32–2.45)	<0.001
Harrell C-statistic	0.62 (0.60–0.64)		0.63 (0.62–0.65)		0.67 (0.65–0.69)	
3-month AUC (95% CI)^b^	0.72 (0.63–0.79)		0.71 (0.62–0.79)		0.71 (0.63–0.79)	
6-month AUC (95% CI)^b^	0.65 (0.59–0.71)		0.67 (0.61–0.73)		0.71 (0.65–0.78)	
iAUC (95% CI)^b^	0.67 (0.61–0.72)		0.69 (0.64–0.73)		0.71 (0.65–0.76)	

^a^NLR50 refers to the median NLR value of our population.

^b^95% CI of AUC(*t*) and iAUC was generated based on 1000 bootstrap replicates.

Similarly, both RMH score and NLR50 (using the median NLR of our population) were also found to significantly predict OS when validating RMH + NLR50 model [12] using the NCIS data (Table 2). The trend increase in the hazard of mortality of RMH as observed in the RMH + NLR50 model, remained significant after adjusting for the effect of NLR50 (HR 1.38, 95% CI 1.08–1.76, *P* = 0.009). However, the addition of NLR50 to the RMH model did not significantly improve the prognostic ability of the RMH score with a similar iAUC of 0.69 (95% CI: 0.64–0.73). The difference in iAUC between these models was 0.018 (95% CI −0.055 to 0.091) (Table 2).

**Fig. 2:**
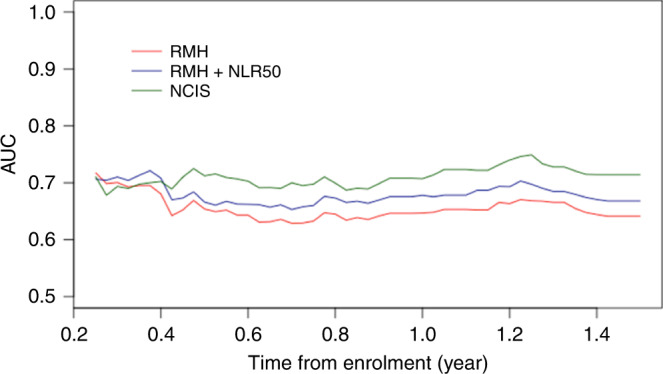
Comparison of AUC(t) of RMH, RMH + NLR50 and NCIS models.

While both RMH and RMH + NLR50 models have fair 3-month AUC, they exhibited a progressive decline in AUC when used to predict the survival status of patients at more distant time points, as evidenced by the lower 6-month AUC and iAUC (Table 2 and Fig. 2).

We hence sought to update the RMH and RMH + NLR50 models by considering the inclusion of other significant variables that were associated with mortality. In our model, ECOG PS 1 was found to portend poorer prognosis than patients with ECOG PS 0 (HR = 1.59 (95% CI: 1.24–2.04), *P* < 0.001) (Table 2). The primary tumour site was also found to be prognostic, with GI primaries (colorectal and upper GI) having poorer OS than breast cancer (HR = 3.06, 95% CI = 2.16–4.35, *P* < 0.001).

### Development of the NCIS Prognostic Score

**Fig. 3:**
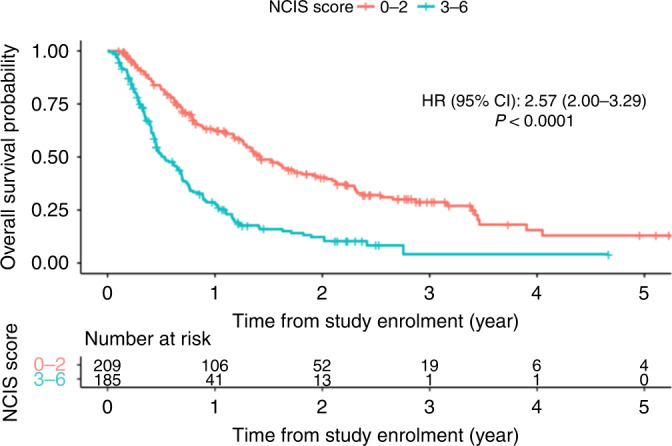
Overall survival according to NCIS Prognostic Score for patients enrolled in Phase I trials.

From the updated NCIS model comprising the following significant predictors (RMH score, ECOG PS and tumour type) (Table 2), we developed a prognostic score as shown in Table 3. We then classified patients into low-risk (score 0–2) and high-risk (score 3–6) groups. The high-risk group had significantly poorer OS compared to the low-risk group (HR 2.57; 95% CI = 2.00–3.29) (Fig. 3).

**Table 3:** NCIS prognostic scoring.

Characteristics	Score
RMH score	
0	0
1	1
2	2
3	3
ECOG PS	
0	0
1	1
Tumour type	
Breast	0
GI malignancy	2
Others	1

The three prognostic models performed similarly in predicting 90-day mortality with AUC ranging between 0.71 and 0.72 (Table 2 and Fig. 2). While substantial declines in AUC were noted for both RMH and RMH + NLR50 models after 90-day, improvements in AUC were observed in the NCIS model from around 5 months onwards and remained relatively stable thereafter (Fig. 2). The NCIS model with AUC at 6-month of 0.71 (95% CI 0.65–0.78) appeared to perform better than both RMH (AUC 0.65, 95% CI 0.59–0.71) and RMH + NLR50 (AUC 0.67, 95% CI 0.61– 0.73) in predicting 6-month mortality, with improvements in AUC of 0.059 (95% CI −0.029 to 0.145) and 0.045 (95% CI −0.045 to 0.135) respectively. Furthermore, its C-index of 0.67 (95% CI 0.65–0.69) and iAUC of 0.71 (95% CI 0.65–0.76) were also higher than the other two models.

## Discussion

Similar to other studies, we found the 90-day mortality rate in Phase I studies in our patient population to be ~17%, but treatment-related deaths were very rare [1, 2]. This demonstrates that poor outcomes in the studies are more related to poor patient selection and cancer progression than grade 5 (fatal) drug adverse events.

Compared to the original RMH score validation cohort [6], our population had a higher proportion of females, patients with breast cancer and patients with better ECOG PS (Table 1). In contrast to the test cohort in the Kumar et al. population [12], our population had a higher proportion of females, and fewer patients with ≥3 metastatic sites. This study also showed results consistent with that of previous studies validating the RMH score [7–10, 18]. As in other studies which have demonstrated the utility of this score in various types of cancers [7, 8, 18], it showed the prognostic score is effective when applied to a population of varied tumour types, and demonstrates broad applications for clinical trials involving all cancers. The RMH model (Model 1) also significantly predicted the 90-day mortality rate. Our data showed that 31% of the patients who died within 90 days had a high RMH score. Using the score would thus help reduce patient attrition during trial recruitment. However, this comes at the trade-off of excluding 17% of total recruitment (17 patients) with high RMH score but with mortality beyond 90 days. We report a median overall survival duration of 10.1 months, consistent with a median survival of 5–10 months reported in other Phase I trials [5, 6, 19–24]. In light of this data and with the advent of novel therapeutics such as immunotherapy and targeted therapy that has less toxicity compared to chemotherapy, a prognostic scoring system to allow prediction of mortality beyond 90 days may be more useful.

Our study is one of the first studies aiming to validate the RMH score in the Asian population participating in Phase 1 studies. Minami et al. [25] demonstrated the utility of the RMH score in predicting progression-free survival (PFS) in a single centre in Japan, but only amongst lung cancer patients in a non-trial setting. In Asia, there are wide variations in the cancer incidence and mortality due to the different ethnic groups and socio-economic status within the region [26] and thus, our study population is representative of this variability. As an academic Phase 1 trial unit which participates in many multi-centre, international Phase I trials, it is important to identify methods to improve and optimise trial participant selection. This would also help investigators to provide patients with a more realistic prognosis to align expectations for their trial participation.

Although other groups have shown the prognostic ability of NLR [12], we observed that the incorporation of NLR in our patient population did not demonstrate additional discriminative ability. RMH and RMH + NLR50 scores includes albumin, LDH and NLR which are laboratory-based biomarkers. While they are useful for predicting 90-day mortality, the models showed limited utility when predicting longer-term OS. The physiology of how higher NLR links to poorer outcomes has always been poorly understood. While tumour-promoting inflammation is recognised as a hallmark of cancer, the association between higher NLR and greater systemic inflammation has only been theorised but never proven. When applied in other studies [12], it showed only modest C-indices in predicting overall survival. The variation in cut-off across different malignancies and study populations as seen in other studies [11, 12] belies the difficulty in finding a cut-off that would be generalisable and easy to use in a general Phase I population. Using a dichotomy for a continuous variable may also result in possible loss of information.

The NCIS score holds its place amongst prognostic scores. The inclusion of variables such as ECOG PS and tumour type does improve the predictive ability of the score. This was confirmed and applied in the MD Anderson Cancer Centre (MDACC) score which also incorporates ECOG PS and tumour type [7], and the Princess Margaret Hospital Index which uses ECOG PS. Whilst they identify GIST as a poor prognostic factor, our group identifies breast cancer as a favourable prognostic factor and GI malignancies as a poor prognostic factor, in our population which had a larger proportion of breast cancer patients as compared to patient populations from other studies [6, 12]. While NLR was not a significant predictor in our NCIS model, it remains used in other models such as the RMH + NLR50 and the Gustave Roussy Score. Thrombocytopenia was also used as a negative prognostic factor in the MD Anderson Immune Checkpoint Inhibitors Score. However, it was not found to be prognostic in our population (data not shown). While hyponatremia was also incorporated into the Hammersmith Score and Nijmegen score, we are not able to validate these models in our population as we did not routinely collect information on sodium levels.

A key strength of this study is the analysis of the prognostic scores’ predictive strengths over time, based on iAUC and AUC(*t*). As we are observing longer median OS in current Phase 1 study participants due to the nature of contemporary treatments such as immunotherapy, prognostic scores to predict survival beyond 90 days may be more meaningful. We observed a decrease in AUC for both RMH and RMH + NLR50 models after 90 days, and improvements in AUC were observed in the NCIS model from around 5 months onwards and remained relatively stable thereafter (Fig. 2). At 6 months, the NCIS model with AUC of 0.71 (95% CI 0.65–0.78) performed better than both RMH (AUC 0.65, 95% CI 0.59–0.71) and RMH + NLR50 models (AUC 0.67, 95% CI 0.61–0.73) in predicting mortality. Furthermore, its iAUC of 0.71 (95% CI 0.65–0.76) was also higher than the other two models. To our knowledge, this is the first study which has identified this limitation of the RMH and RMH + NLR50 models.

As previously mentioned, another strength of the paper is that this is the first paper to validate the RMH score in a population of Asian Phase I patients, and one of the largest validation studies of Phase I patients in general. The NCIS prognostic score is also developed in patients on more contemporary treatments such as immunotherapy and vaccine therapy. While additional variables may be more challenging to apply in clinical practice, such information is routinely obtained and unlikely to be a hindrance. It would also be very possible to incorporate automated calculation onto electronic clinical records platforms to facilitate decision making, thus meaningful to validate this score prospectively.

There are however limitations to this study. As a retrospective analysis, it is subject to selection bias. We attempted to minimise bias by having clear inclusion criteria and including all patients who met the inclusion criteria within the time frame of analysis. This resulted in a broad sample of various cancer types and histologies. We also recognise the varying prognosis of different cancers from the point of diagnosis, with the rapid progression of cancer therapy. Thus including all cancer types might appear to result in a highly heterogeneous population. The target population of our study however are cancer patients being considered for Phase I clinical trials, who are usually heavily pre-treated and have exhausted standard therapy. The outcome measure is survival from point of first visit, which is more homogeneous amongst different cancer types than overall survival from the point of diagnosis. Furthermore, a prediction of 90-day mortality from the time of study enrolment is a standard inclusion criterion for Phase I trials. Phase 1 studies consist of the basket and non-basket trials. Thus having a simple score that applies to all tumour types and different Phase 1 trial designs allow for easier and hence broader uptake at the point of the first visit. This is also a single-centre study with a modest sample size, and would require additional studies before clear conclusions can be made of the Asian oncology population at large. The limited sample size also resulted in inadequate power to establish statistical significance in the comparison of AUC(*t*) and iAUC amongst the different prognostic models. However, a 4–6 percentage point improvement in AUC over established models is considerable in validation studies, suggesting that further large prospective studies should be conducted to validate our findings and confirm the superiority of the NCIS score.

## Conclusion

In summary, the original RMH score is a useful adjunct to identify patients with poor prognoses to reduce patient attrition in Phase I studies in the Asian population, but its predictive ability decreases when predicting longer-term survival beyond 90 days. The addition of NLR did not improve the predictive ability of prognostic scores. Our NCIS score provides the excellent discriminatory ability for both short-term and longer-term survival in patients on contemporary Phase I studies and prospective studies should be conducted to validate the NCIS score in different Phase I patient populations.

## Data Availability

The datasets generated during and/or analysed during the current study are available from the corresponding author on reasonable request.
